# Investigating the Role of Serum and Plasma IL-6, IL-8, IL-10, TNF-alpha, CRP, and S100B Concentrations in Obstructive Sleep Apnea Diagnosis

**DOI:** 10.3390/ijms241813875

**Published:** 2023-09-09

**Authors:** Piotr Fiedorczuk, Ewa Olszewska, Agnieszka Polecka, Marzena Walasek, Barbara Mroczko, Agnieszka Kulczyńska-Przybik

**Affiliations:** 1 Department of Otolaryngology, Medical University of Bialystok, 15-089 Bialystok, Poland; piotr.fiedorczuk@sd.umb.edu.pl (P.F.); mnwalasek@gmail.com (M.W.); 2 Doctoral School of the Medical, University of Bialystok, 15-089 Bialystok, Poland; agnieszka.polecka@sd.umb.edu.pl; 3 Department of Neurodegeneration Diagnostics, Medical University of Bialystok 15-089 Bialystok, Poland; mroczko@umb.edu.pl (B.M.); agnieszka.kulczynska-przybik@umb.edu.pl (A.K.-P.)

**Keywords:** obstructive sleep apnea, biomarkers, plasma and serum concentrations

## Abstract

Obstructive sleep apnea (OSA) is a prevalent and underdiagnosed condition associated with cardiovascular diseases, depression, accidents, and stroke. There is an increasing need for alternative diagnostic tools beyond overnight sleep studies that measure the Apnea/Hypopnea Index (AHI). In this single-center, case-control study, we evaluated serum and plasma concentrations of IL-6, IL-8, IL-10, TNF-α, CRP, and S100B in 80 subjects, including 52 OSA patients (27 moderate [15 ≤ AHI ˂ 30], 25 severe [AHI ≥ 30]) and 28 non-OSA controls (AHI 0-5). Participants with OSA showed approximately 2 times higher median concentrations of CRP in plasma, and IL-6 in serum, as well as 1.3 to 1.7 times higher concentrations of TNF-α and IL-8 in plasma compared with the control group. Receiver Operator Characteristic (ROC) curve analysis was performed to evaluate the predictive capabilities of these serum and plasma biomarkers in distinguishing between the OSA and control groups, revealing varying sensitivity and specificity. In summary, in this study, serum and plasma biomarkers CRP, S100B, IL-6, TNF-α, and IL-8 have been shown to be elevated in patients with OSA, correlated positively with disease severity, age, and BMI. These results support the potential role of these biomarkers in diagnosing OSA, supplementing traditional methods such as overnight sleep studies.

## 1. Introduction

Obstructive Sleep Apnea Syndrome (OSA) is a disorder with a high prevalence, estimated at 9% to 38% in the general adult population, 13–33% in men, and 6–19% in women [1]. Up to 90% of individuals with OSA remain undiagnosed or without therapy, leaving them at an increased risk for hypertension, cardiovascular disease, heart failure, obesity, metabolic imbalance, diabetes mellitus, excessive daytime sleepiness, depression, accidents, and stroke [2,3,4,5]. The pathogenesis of OSA is multifactorial and still not fully identified. It involves various mechanisms including selective activation of inflammatory pathways, endothelial dysfunction, metabolic dysregulation, and oxidative stress [6,7,8]. The potential origins of OSA can be attributed to several factors, encompassing anatomical considerations, impaired functionality of the dilator muscles, respiratory control instability characterized by a high loop gain, and a lowered threshold for arousal [9]. Endothelial dysfunction is often regarded as one of the earliest detectable and potentially reversible abnormalities during the development of atherosclerosis [10].

According to current practice guidelines for OSA, there is a need for the development of novel clinical tools to better assess the disease burden [2]. Overnight polysomnography (PSG) holds a well-established reputation as the definitive ‘gold standard’ for diagnosis. In a parallel vein, the precise localization of anatomical obstructions finds its resolution through the utilization of Drug-Induced Sleep Endoscopy (DISE). It is important to note, however, that this is an invasive method [9]. Innovative screening and diagnostic methods that surpass the limitations of the conventional overnight sleep study and DISE may simplify the diagnostic process and allow for earlier treatment interventions, for instance, positive airway pressure (PAP) therapy, thereby preventing the onset of serious comorbidities. The quantification of inflammatory cytokines, markers of endothelial dysfunction, and proteins linked to intermittent hypoxia in serum or plasma, may aid in OSA screening, prognosis, diagnosis, and monitoring of the effectiveness of treatment [11,12]. Based on available literature data, interleukin-6 (IL-6), interleukin-8 (IL-8), interleukin-10 (IL-10), tumor necrosis factor-alpha (TNF-α), C-reactive protein (CRP), and neuronal injury markers like S100 calcium-binding protein B (S100B) could potentially play a role in OSA management [12,13,14,15,16].

IL-6, a cytokine with multiple functions, is widely acknowledged to mediate the acute phase response. Apart from its involvement in host defense, inflammation, and cancer, IL-6 is also believed to contribute to cell proliferation and hypertrophy [17]. Vascular cell-derived IL-6 has been linked to various stimuli, including inflammatory cytokines and growth factors, which can increase IL-6 production. Other factors such as cigarette smoke and adiposity can also induce its expression. Elevated levels of IL-6 have been observed in healthy individuals experiencing hypoxemia at high altitudes [18,19]. Obesity leads to heightened levels of IL-6 in both serum and plasma [20]. Notably, obesity stands as the primary risk factor for OSA [9]. As a result, the amplified IL-6 presence, while pertinent, assumes a confined role within the context of OSA. IL-6 levels are often elevated in cardiovascular diseases like atherosclerosis and hypertension and are thought to drive the structural and functional changes in arteries due to these conditions [21].

IL-8, a chemoattractant cytokine, is generated by various tissue and blood cells, with mononuclear macrophages being the primary source. When suitably stimulated, epithelial and endothelial cells can also produce IL-8 [22]. In inflamed areas, IL-8 attracts and activates neutrophils, sustaining inflammation by inducing neutrophils to generate myeloperoxidase and attract other inflammatory cells. IL-8’s interaction with specific receptors on neutrophils triggers cell deformation, degranulation, and increased production of reactive oxygen species. This process can lead to the release of lysosomal contents, activation of arachidonic acid, and enhanced vascular permeability and plasma protein exudation, ultimately resulting in tissue damage, atherosclerosis, vascular inflammation, and other disorders [23]. OSA patients may exhibit rapid macrophage antigen 1 mobilization to the neutrophil surface upon exposure to IL-8. Consequently, IL-8 upregulation has been observed in human bronchial epithelial cells in response to vibration stimulation caused by snoring [24]. IL-8 is involved in physiological sleep regulation in healthy individuals and is associated with physiological secretory patterns [25]. Lower IL-8 secretion corresponds to better nighttime sleep and a healthier state the following day, while higher IL-8 secretion may be linked to excessive daytime sleepiness and fatigue [26].

IL-10 is an anti-inflammatory cytokine with immunomodulatory properties that plays a significant role in the regulation of the immune response. IL-10 is known to counterbalance the effects of proinflammatory cytokines, such as IL-6 and TNF-α, by inhibiting their production and promoting the resolution of inflammation [27]. In the context of OSA, IL-10 may serve as a protective factor against excessive inflammation and its associated complications. Studies have suggested that IL-10 levels may be altered in patients with OSA, potentially indicating an imbalance in the inflammatory response. Furthermore, IL-10 has been associated with the severity of OSA, with lower levels observed in more severe cases [28]. This relationship suggests that IL-10 could potentially serve as a diagnostic biomarker for OSA, helping to identify and stratify patients according to disease severity.

CRP is a crucial blood marker of inflammation that is produced in the liver and primarily regulated by the proinflammatory cytokine interleukin-6 [29]. Unlike other cytokines, CRP levels remain relatively consistent within an individual over a 24 h period, which can help indicate the magnitude of the inflammatory response. Epidemiological studies have proven high-sensitivity CRP to be an independent risk factor for cardiovascular diseases [30]. Elevated CRP levels are associated with future cardiovascular events in patients with stable angina pectoris, acute coronary artery disease, and prior myocardial infarction. CRP may play a direct role in initiating and promoting atherosclerosis. CRP contributes to the proinflammatory and proatherogenic characteristics of endothelial cells, vascular smooth muscle cells, and monocyte macrophages by influencing processes such as cell adhesion, cytokine production, and cell proliferation. This suggests a potential link between elevated CRP levels and increased cardiovascular risk in OSA patients [31].

TNF-α is a proinflammatory cytokine essential in host defense and implicated in the pathogenesis of numerous diseases, such as atherosclerosis, inflammatory bowel disease, and autoimmune disorders [32].

The initial discovery of TNF-alpha within tumor necrosis substantiates its nomenclature. This marker exhibits augmented concentrations across a spectrum of cancerous conditions. This correlation underscores its potential significance as a diagnostic indicator and therapeutic target in oncology. It is involved in several necrosis- and apoptosis-related signaling pathways and has a regulatory role in sleep, being associated with excessive daytime sleepiness, disrupted nighttime sleep, and hypoxia [33].

S100B, a calcium-binding protein primarily found in glial cells, has been implicated in various physiological and pathological processes in the central nervous system Elevated S100B levels are associated with issues like brain injuries, strokes, infections, and neurodegenerative disorders. Emerging evidence suggests a potential link between S100B and OSA, with alterations in its levels observed in patients with the disorder [15]. The role of S100B in OSA may be related to its involvement in inflammation, oxidative stress, and neuronal damage, which are known contributors to the pathophysiology of OSA. Consequently, S100B could serve as a potential biomarker for OSA diagnosis, offering insights into disease severity and aiding in the identification of patients who may benefit from treatment interventions.

Most studies available to date have reported the concentrations of IL-6, IL-8, IL-10, TNF-α, CRP, or S100B in one cell-free blood fraction, serum, or plasma [34]. During the coagulation process for serum collection, proteins and metabolites are released by platelets into the serum. Contrarily, plasma is initially treated with anticoagulants (such as ethylenediaminetetraacetic acid (EDTA), heparin, and citrate) before the blood cells are separated by centrifugation, ensuring that the liquid still contains deactivated clotting components. These processes can cause alterations in the metabolome or proteome and affect the concentrations of IL-6, IL-8, IL-10, TNF-α, CRP, and S100B in the serum and plasma. To our knowledge, no study attempted to compare the diagnostic capability of IL-6, IL-8, IL-10, TNF-α, CRP, and S100B between the concentrations in the serum and plasma in OSA patients before the treatment.

Laboratory tests offer a multifaceted advantage, providing a non-invasive, easily attainable, and economical approach to assessment, while also playing a pivotal role in diagnosing various medical conditions within routine clinical practice, with notable emphasis on screening procedures and accurate disease diagnostics. Diagnostic biomarkers, including specific molecules, genes, or other biological substances, are critical tools in modern medicine, aiding in the early detection, diagnosis, and management of diseases [35]. They offer objective, quantifiable information that assists clinicians in making informed decisions about patient care, enabling insights into underlying pathology, early disease identification, monitoring illness progression, and assessing treatment effectiveness. Additionally, the development of novel diagnostic biomarkers has the potential to enhance diagnostic accuracy, reduce the need for invasive procedures, and guide personalized treatment plans. As a result, ongoing research in this area continues to shape the landscape of modern medicine, with the ultimate goal of improving patient outcomes and enhancing overall healthcare quality.

In this context, our study aimed to determine the potential utility of potential biomarkers, such as IL-6, IL-8, IL-10, TNF-α, CRP, and S100B, in the diagnostic process of obstructive sleep apnea (OSA). We compared the levels of these biomarkers in serum and plasma among a single patient cohort, which included moderate and severe OSA patients as well as non-OSA control subjects. Furthermore, we assessed whether the concentrations of these biomarkers in serum differed from those in plasma and evaluated which demonstrated superior predictive capacity for OSA diagnosis.

## 2. Results

In our study, we recruited a total of 80 participants, consisting of 52 individuals diagnosed with moderate (15 ≤ AHI ˂ 30) and severe (AHI ≥ 30) OSA, and 28 control subjects without OSA. The basic demographic, anthropometric, and sleep test parameters and comorbidities of the study participants are presented in Table 1.

As we can observe in Table 1, the majority of participants in both the OSA and control groups were male, aand no significant differences between groups were observed regarding genders. Both groups were matched for age and sex, but the OSA group exhibited a significantly higher body mass index (BMI) compared with the control group. The OSA group also showed a significantly higher AHI, which served as the criterion for assigning participants to their respective groups. Both the OSA and control groups showed the presence of hypertension, with a significantly higher prevalence observed in the Severe OSA group. Although the OSA group had a higher proportion of participants with comorbidities including diabetes, coronary artery disease, and hyperuricemia compared with the control group, the difference did not reach statistical significance. Additionally, statistically significant differences were observed in MOS, LSAT, and SpO2 < 90% between the OSA and control groups, as well as between the Moderate and Severe OSA groups and the control group (all *p* < 0.0001).

**Figure 1:**
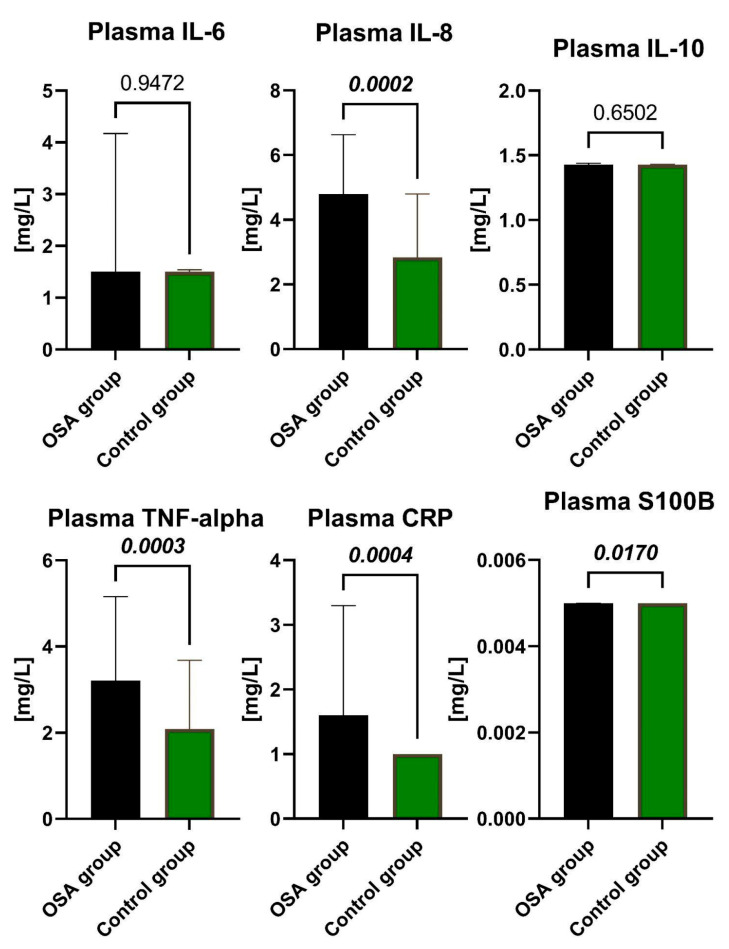
The concentrations of IL-6, IL-8, IL-10, TNF-α, CRP, and S100B in the plasma of the participants from the OSA group and Control group. Data represent the median and interquartile range and *p*-value of the OSA group vs. the Control group. The Mann–Whitney U test was utilized for comparisons across the groups, with *p* < 0.05 considered statistically significant. Abbreviations: IL-6, Interleukin-6; IL-8, Interleukin-8; IL-10, Interleukin-10; CRP, C-reactive protein; TNF, Tumor Necrosis Factor; S100B, S100 calcium-binding protein B.

Patients from the OSA group were divided into two subgroups based on AHI—28 patients with AHI ≥ 15 but < 30 into the Moderate OSA group, and 25 patients with AHI ≥ 30 into the Severe OSA group. We observed that participants with OSA in the study group had approximately 2 times higher median concentrations of CRP in plasma compared to the non-OSA control group (*p* = 0.0004). The presented results are depicted in Figure 1 below. Similarly, the concentrations of TNF-α in plasma were about 1.5 times higher (*p* = 0.0003), and the concentrations of IL-8 in plasma were nearly 1.7 times higher (*p* = 0.0002) in the study group compared with the control group. All of the above-mentioned differences between the OSA and non-OSA groups reached statistical significance. For some biomarkers, namely IL-6 IL-10, CRP in serum and IL-6, IL-10, CRP, TNF-α, and S100B in plasma, the median concentration was below the detection threshold in one or both groups, indicating that at least half of the values were below this threshold.

Regarding serum levels, the study group had approximately 2 times higher median concentrations of CRP (*p* = 0.0002) in serum and IL-6 (*p* = 0.0006) in serum compared with the control group. Additionally, the concentration of TNF-α (*p* = 0.0095) in serum was about 1.3 times higher in the OSA group than in the control group. All of the above-mentioned differences between the OSA and control groups reached statistical significance. Notably, there were no statistically significant differences between the groups for the concentrations of IL-10 in both serum and plasma. For some biomarkers in serum, the median concentrations were below their respective detection thresholds in one or both groups.

**Figure 2:**
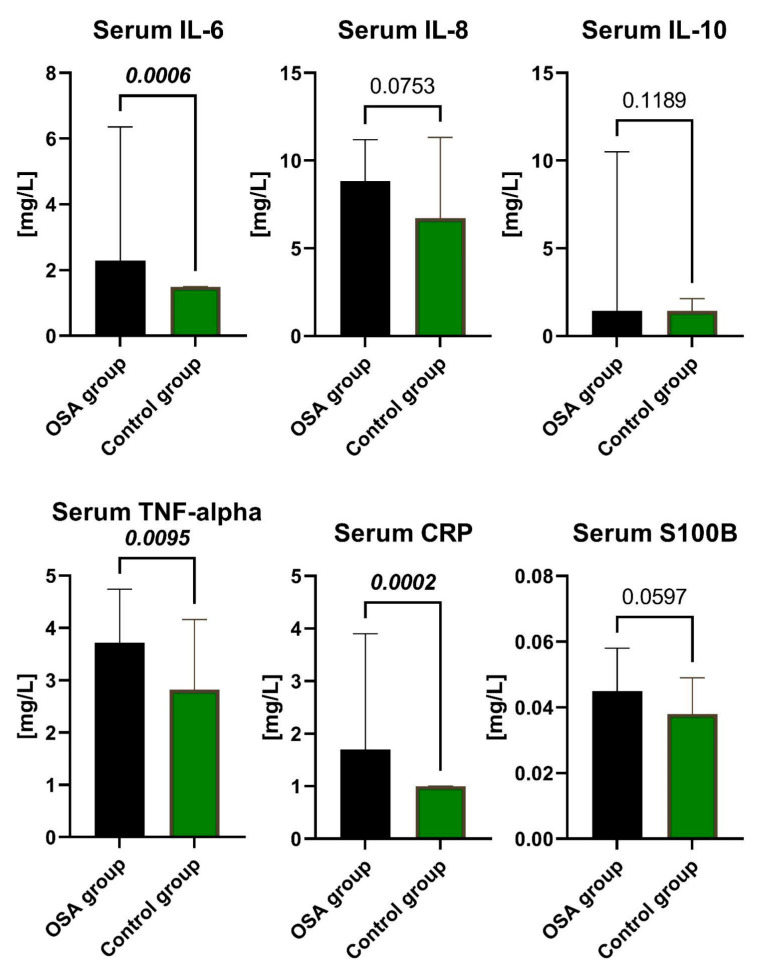
The concentrations of IL-6, IL-8, IL-10, TNF-α, CRP, and S100B in the serum of the participants from the OSA group and Control group. Data represent the median and interquartile range and *p*-value of the OSA group vs. the Control group. The Mann–Whitney U test was utilized for comparisons across the groups, with *p* < 0.05 considered statistically significant. Abbreviations: IL-6, Interleukin-6; IL-8, Interleukin-8; IL-10, Interleukin-10; CRP, C-reactive protein; TNF-α, Tumor Necrosis Factor; S100B, S100 calcium-binding protein B.

Comparisons between the study and control groups are shown in Figure 1 and Figure 2. Calculated medians, minimum-maximum values, and interquartile ranges of the concentrations of IL-6, IL-8, IL-10, TNF-α, CRP, and S100B in serum and plasma, along with the effect size of calculations, are presented in the Supplementary Material—Table S1.

**Figure 3:**
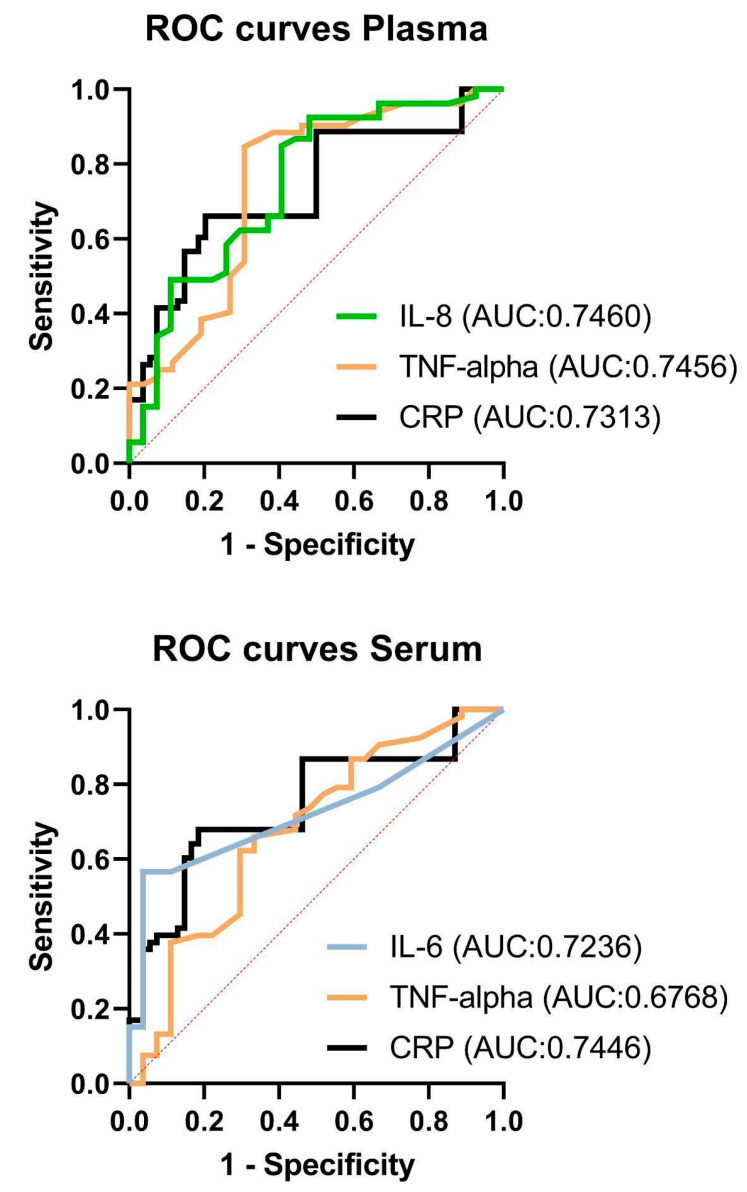
ROC curves of the concentrations of IL-6, IL-8, IL-10, TNF-α, CRP, and S100B in the plasma and serum. The Receiver Operator Characteristic curve analysis is calculated for all subjects in classifying for OSA and non-OSA groups. Abbreviations: IL-6, Interleukin-6; IL-8, Interleukin-8; CRP, C-reactive protein; TNF, Tumor Necrosis Factor; S100B, S100 calcium-binding protein B ROC, Receiver Operator Characteristic; AUC, Area Under the Curve.

Receiver Operator Characteristic (ROC) curves were computed to assess the predictive capabilities of various serum and plasma biomarkers in distinguishing between the OSA group and the Control group, as depicted in Figure 3. The evaluated biomarkers included CRP, IL-8, TNF-α, and S100B in plasma, as well as IL-6, TNF-α, and CRP in serum. These ROC curves illustrate the trade-off between sensitivity and specificity across different discrimination thresholds.

In terms of the ROC analysis results, each biomarker’s Area Under the Curve (AUC) and *p*-values were computed to quantify their discriminatory power:

- Plasma CRP (AUC = 0.7313, *p* = 0.0008)
- Plasma IL-8 (AUC = 0.7460, *p* = 0.0003)
- Plasma TNF-α (AUC = 0.7456, *p* = 0.0004)
- Plasma S100B (AUC = 0.6073, *p* = 0.1183)
- Serum IL-6 (AUC = 0.7236, *p* = 0.0011)
- Serum TNF-α (AUC = 0.6768, *p* = 0.0100)
- Serum CRP (AUC = 0.7446, *p* = 0.0004)

Measured potential biomarkers demonstrated varying sensitivity and specificity in predicting the presence of moderate and severe OSA (AHI ≥ 15) at their respective cut-off values. The Likelihood Ratio (LR) measures the effectiveness of a diagnostic test. A positive LR is the ratio of the true positive rate (sensitivity) to the false positive rate (1—specificity). A higher LR indicates better diagnostic performance, with a value above 10 typically considered strong evidence for the presence of the target condition.

The calculated results for sensitivity, specificity, and LR were:

- Plasma CRP—sensitivity 26.42%, specificity 96.30%, and LR 7.132;
- Plasma IL-8—sensitivity 33.96%, specificity 92.59%, and LR 4.585;
- Plasma TNF-α—sensitivity 21.15%, specificity 96.15%, and LR 5.500;
- Plasma S100B—sensitivity 98.11%, specificity 0.00%, and LR 0.9811;
- Serum IL-6—sensitivity 56.60%, specificity 96.30%, and LR 15.28;
- Serum TNF-α—sensitivity 37.74%, specificity 88.89%, and LR 3.396;
- Serum CRP—sensitivity 35.85%, specificity 96.30%, and LR 9.679.

Among the plasma and serum parameters, most values exhibited higher specificity and lower sensitivity, except for S100B in plasma. This suggests that these markers tend to accurately identify the Control group but might be less sensitive in detecting the OSA group.

A combination of potential serum and plasma biomarkers—IL-6, IL-8, TNF-α, CRP, and S100B—was tested for predicting classification into OSA and non-OSA subjects. Multiple logistic regression models including the concentrations of IL-8, TNF-α, CRP, and S100B in the serum and IL-6, TNF-α, and CRP in plasma showed that only CRP and IL-6 were a non-zero estimate (CRP 95% CI 0.06182 to 1.377, TNF-α 95% CI 0.03904 to 0.8016 in the serum, CRP in the plasma 95% CI 0.08746 to 1.585), while IL-8 and S100B were not significant. The results of the combined model suggest that among the tested biomarkers, CRP and IL-6 are statistically significant predictors of this classification. This indicates that the concentrations of CRP and IL-6 play a role in differentiating between individuals with OSA and those without. However, the model does not necessarily indicate the sole use of CRP and IL-6 for diagnosis or prediction. Instead, it highlights their importance in the context of this specific study and dataset.

**Figure 4:**
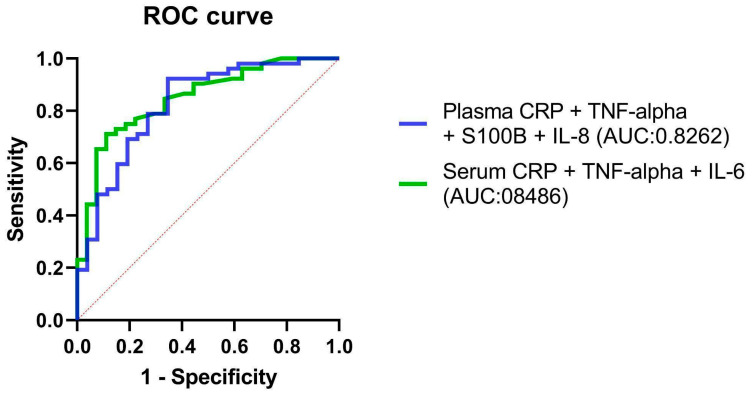
ROC curves of the combined concentrations of IL-8, TNF-α, CRP, and S100B in the serum and IL-6, TNF-α, and CRP in plasma. Abbreviations: IL-6, Interleukin-6; IL-8, Interleukin-8; CRP, C-reactive protein; TNF, Tumor Necrosis Factor; S100B, S100 calcium-binding protein B; ROC, Receiver Operator Characteristic; AUC, Area Under the Curve.

The ROC curves of combined IL-8, TNF-α, CRP, and S100B in the serum and IL-6, TNF-α, and CRP in plasma models are shown in Figure 4.

Numerous positive or negative correlations were noted between the investigated parameters. Table 2 displays the results of Spearman correlation analysis, illustrating the relationships between various biomarkers’ concentrations in both serum and plasma, as well as their correlations with OSA severity, sex, age, BMI, and AHI.

The study reveals significant correlations involving IL-6 in serum. Positive associations are found with IL-10, CRP, S100B, and TNF-alpha concentrations in serum, as well as IL-6 concentrations in plasma. These correlations extend to factors like OSA status, age, BMI, and AHI.

Regarding IL-6 plasma concentrations, significant positive correlations emerge with IL-6 and IL-8 concentrations in serum, CRP concentrations in serum, IL-8 concentrations in plasma, CRP concentrations in plasma, S100B concentrations in plasma, and TNF-alpha concentrations in plasma.

Similarly, the concentrations of IL-8 in serum exhibit a significant positive correlation with both IL-8 concentrations in plasma and BMI.

IL-10 concentrations in serum align with IL-6, S100B, and TNF-alpha concentrations in serum, as well as IL-10 concentrations in plasma.

CRP concentrations in serum show significant positive correlations with IL-6 concentrations in both serum and plasma, IL-8 concentrations in plasma, and TNF-alpha concentrations in plasma. S100B concentrations in serum connect positively with IL-6 concentrations in serum and IL-10 concentrations in serum, and negatively with S100B concentrations in plasma. TNF-alpha concentrations in serum correlate positively with IL-6 and IL-10 concentrations in serum, IL-8 concentrations in plasma, and TNF-alpha concentrations in plasma, as well as OSA status, BMI, and AHI.

IL-8 concentrations in plasma show significant positive correlations with IL-8 concentrations in serum, CRP concentrations in serum, TNF-alpha concentrations in serum, IL-6 concentrations in plasma, and TNF-alpha concentrations in plasma. These concentrations also exhibit associations with OSA status, BMI, and AHI. CRP concentrations in plasma demonstrate positive connections with IL-6 concentrations in serum, CRP concentrations in serum, TNF-alpha concentrations in serum, IL-6 concentrations in plasma, and TNF-alpha concentrations in plasma, as well as OSA status, age, BMI, and AHI. S100B concentrations in plasma exhibit both positive and negative correlations, connecting with IL-6 concentrations in serum, IL-6 concentrations in plasma, OSA status, and AHI.

Moreover, TNF-alpha concentrations in plasma are positively linked to IL-6 concentrations in serum, CRP concentrations in serum, TNF-alpha concentrations in serum, IL-6 concentrations in plasma, IL-8 concentrations in plasma, CRP concentrations in plasma, OSA status, BMI, and AHI. Remarkably, OSA status is associated with a range of variables, showing positive correlations with IL-6 and CRP concentrations in serum, TNF-alpha concentrations in serum, IL-8 concentrations in plasma, CRP concentrations in plasma, S100B concentrations in plasma, and TNF-alpha concentrations in plasma, as well as sex, BMI, and AHI. Additionally, sex correlates with OSA status and AHI. Age demonstrates positive correlations with IL-6 concentrations in serum, IL-8 concentrations in plasma, CRP concentrations in plasma, BMI, and AHI. Lastly, BMI’s positive correlations span IL-6 and IL-8 concentrations in serum, CRP concentrations in serum, TNF-alpha concentrations in serum, IL-8 concentrations in plasma, TNF-alpha concentrations in plasma, OSA status, and AHI. These associations highlight the intricate interrelationships among these variables in the study’s context.

However, not all biomarkers showed this positive correlation with AHI. Non-significant correlations were observed for IL-8 in serum, IL-10 in serum, S100B in serum, IL-6 in plasma, IL-10 in plasma, S100B in plasma, and IL-8 plasma with AHI. These biomarkers may not be strongly linked to OSA severity in this study. These differential correlation patterns underscore the complexity of the biomarker landscape in OSA and the distinct roles of individual biomarkers in reflecting disease severity.

### Moderate vs. Severe OSA Comparison

Based on AHI, we categorized the subjects into Moderate OSA and Severe OSA groups. Despite observed variations in biomarker levels between these groups, it is crucial to highlight that no significant differences were found, as reflected by the provided *p*-values.

Though subtle variations could be observed in the median concentrations of certain plasma biomarkers like CRP, TNF-alpha, and IL-8, with the Severe OSA group seemingly presenting higher levels compared to the Moderate OSA group, these differences did not achieve significance. This was also the case for IL-6 and IL-10, where minimal variations between the groups were observed. In the context of serum biomarkers, despite the Severe OSA group displaying marginally higher median concentrations of CRP and S100B and a slightly lower level of IL-6, these differences were not significant. The data including above-mentioned serum and plasma biomarkers is presented in Table 3.

Multiple linear regression analyses were conducted to account for potential confounding factors. We scrutinized the correlation between the concentration of the biomarkers that showed a difference in the OSA and Control groups, namely IL-8, TNF-alpha, S100B, and CRP in plasma and IL-6, TNF-alpha, and CRP in serum, and potential confounders such as BMI, age, and gender. In this analysis, the dependent variables were the biomarker levels, and the main independent variable was the OSA status, while the potential confounders were BMI, age, and sex. The results revealed significant associations: the OSA status coefficient showed significance concerning TNF-alpha and IL-8 concentrations in plasma. Additionally, age demonstrated significance concerning IL-6 concentration in plasma. Results are shown in Table 4.

## 3. Discussion

The research provided further evidence for IL-6, IL-8, IL-10, TNF-α, CRP, and S100B to be considered potential candidates for biomarkers for diagnosing or screening for moderate and severe OSA.

The present investigation is unique in its comprehensive analysis of IL-6, IL-8, IL-10, TNF-α, CRP, and S100B in a single cohort of sleep apnea patients compared to non-OSA controls in both serum and plasma. The diagnostic capabilities of IL-6, IL-8, IL-10, TNF-α, CRP, and S100B are evaluated and compared in both serum and plasma. These biomarkers are observed at significantly higher concentrations in individuals with OSA and demonstrate a positive correlation with the severity of the condition, as well as with factors like age and BMI.

The role of IL-6, IL-8, IL-10, TNF-α, CRP, and S100B as potential biomarkers in OSA has been previously investigated in separate studies which were compiled in a recent umbrella review of meta-analyses authored by the investigators of this study [36]. The review found fourteen sets of biomarkers which were identified as candidates and exhibited differences in levels or concentrations between individuals with OSA and non-OSA controls. These biomarkers, including CRP, IL-6, TNF-α, IL-8, homocysteine (HCY), intercellular adhesion molecule-1 (ICAM-1), vascular cell adhesion molecule-1 (VCAM-1), vascular endothelial growth factor (VEGF), total cholesterol (TC), low-density lipoprotein cholesterol (LDLc), high density lipoprotein cholesterol (HDLc), triglycerides (TG), leptin, malondialdehyde (MDA), alanine transaminase (ALT), aspartate aminotransferase (AST), insulin-like growth factor 1 (IGF-1), adiponectin, and cortisol, were found to decrease after OSA treatment. This suggests that these biomarkers may serve as indicators for monitoring the effects of OSA treatment.

A study by Imani et al. reported that plasma CRP levels were significantly higher in OSA patients than in control subjects and were positively correlated with AHI [37]. Similarly, a study by Nadeem et al. found increased serum levels of IL-6 and TNF-α in OSA patients compared with controls [24]. Dogan et al. reported that OSA patients had higher plasma IL-6 levels than control subjects [38]. These findings align with our study, where we observed higher median concentrations of CRP, IL-6, IL-8, and TNF-α in both plasma and serum in the OSA group compared with the control group.

In contrast, our study found no differences between the groups regarding the concentrations of IL-10 in both serum and plasma. This is in line with a study by Yi et al., where they reported no significant difference in the serum IL-10 levels between OSA patients and control subjects [39]. However, other studies have reported contradictory results. For instance, a study by Ryan et al. found that plasma IL-10 levels were significantly higher in OSA patients than in controls [40]. The role of anti-inflammatory IL-10 in OSA diagnosis needs more investigations, perhaps in bigger populations.

The current study is unique in its assessment of IL-6, IL-8, IL-10, TNF-α, CRP, and S100B in both serum and plasma obtained from the blood of OSA and control participants. Previous studies have assessed these biomarkers in either serum or plasma, but not in both, which may have led to inconsistent results. For instance, a study by Gopalakrishnan et al. found increased levels of IL-6 and TNF-α in the serum of OSA patients compared with controls [41], while Vgontzas et al. reported similar findings for plasma IL-6 levels [42]. In our study, we observed a strong positive correlation between the concentrations of the biomarkers in both serum and plasma. This suggests that the choice of serum or plasma may have significant implications for biomarker measurement, and future studies should consider assessing these biomarkers in both serum and plasma for a more comprehensive understanding of their role in OSA.

Differences in serum and plasma concentrations stem from varied sample processing methods, impacting biomarker stability and interactions. Platelet activity and clotting factors in serum may affect biomarker levels differently. Contaminants and individual variations also contribute to disparities, highlighting the need for standardized procedures. Studying both plasma and serum is crucial due to their distinct compositions. Plasma contains coagulation factors, while serum lacks them. This distinction allows researchers to capture a wider range of biomarkers and understand disease mechanisms comprehensively. The choice between plasma and serum affects biomarker stability and accuracy, with diseases potentially influencing levels differently in each. Both sample types offer unique insights, aiding the identification of diagnostic and therapeutic markers.

Our study found that certain biomarkers, such as CRP, IL-6, IL-8, and TNF-α, demonstrated varying sensitivity and specificity in predicting the presence of moderate and severe OSA. When discussing sensitivities and specificities, it is notable that both serum and plasma biomarkers consistently exhibited greater specificity alongside lower sensitivity values. This pattern suggests that these biomarkers tend to more accurately identify true negative cases while potentially being less sensitive in detecting true positives. To provide practical context, specificity reflects the proportion of correctly identified negative cases out of all actual negatives, indicating a low false positive rate. Sensitivity, on the other hand, signifies the proportion of accurately identified positive cases out of all actual positives, illustrating the biomarker’s ability to avoid false negatives. These findings indicate a possible role of these biomarkers in supplementing our understanding of OSA, which could eventually assist in identifying individuals at high risk. However, more comprehensive studies are necessary to affirm their use as minimally invasive diagnostic tools for OSA. Utilizing biomarkers as a diagnostic tool can provide a cost-effective, non-invasive, and accessible method to identify OSA patients, particularly in resource-limited settings. Moreover, it can help reduce the burden on healthcare systems by minimizing the need for polysomnography, an expensive and resource-intensive procedure.

S100B is secreted by various cell types and can be found in multiple bodily fluids. While serum and plasma are closely related, they differ in their content, which could potentially influence S100B levels. We suspect the observed negative correlation might be related to differential protein binding or the clearance rate of S100B in plasma versus serum.

Additionally, the process of coagulation, which separates serum from whole blood, might influence S100B measurements. Platelets, which are present in whole blood and plasma but absent in serum, have been reported to absorb and release S100B. This dynamic could lead to lower S100B concentrations in serum compared with plasma, contributing to the negative correlation that we observed.

Our ROC curve analysis demonstrated that the AUC for the biomarkers CRP, IL-8, and TNF-α in plasma, and IL-6 and CRP in serum, were all above 0.7, underscoring their robust diagnostic performance. Notably, IL-6 in serum exhibited the highest likelihood ratio, thereby confirming its strong potential for OSA detection. However, it is important to highlight that the sensitivity and specificity of individual biomarkers varied significantly. For instance, Plasma S100B displayed high sensitivity but lacked specificity. This indicates that the use of a single biomarker may not provide sufficient diagnostic accuracy for OSA. Interestingly, our logistic regression model indicated that, among a combination of biomarkers, only CRP and IL-6 offered non-zero estimates, thereby hinting at their importance in OSA detection. This leads us to infer that a combined application of select biomarkers might present a more reliable diagnostic method for OSA than any individual biomarker. Therefore, future research should be focused on multi-center studies of larger scale to determine the optimal cut-off points for these biomarkers and tailor these to individual patient profiles such as age, sex, and comorbidity status. This elaboration offers an enriched understanding of our results and charts out potential avenues for the application of biomarkers in the diagnosis of OSA.

The potential for early detection of moderate and severe OSA cases through biomarkers might provide avenues for expedited intervention and management, which could possibly contribute to mitigating the risk of related comorbidities such as hypertension, cardiovascular diseases, and metabolic disorders. This approach might enhance patient outcomes, improve quality of life, and reduce healthcare costs in the long run. Moreover, tracking the response to treatment by observing these biomarkers could be a tool in evaluating the effectiveness of various therapeutic approaches, potentially providing healthcare professionals with insights to modify interventions if required.

### Study Limitations

Our study has several strengths, including a well-defined patient population, the use of a case-control design, and the measurement of a comprehensive panel of biomarkers using a sensitive and specific method (Luminex assay). However, there are also some limitations that should be acknowledged. Firstly, the sample size was relatively small, which may limit the generalizability of our findings. Additionally, the study population consisted mainly of male participants, which may not reflect the full spectrum of OSA patients. Further research with larger and more diverse cohorts is needed to validate our findings and explore possible sex-specific differences in biomarker concentrations.

Secondly, while our study focused on IL-6, IL-8, IL-10, TNF-α, CRP, and S100B, there may be other potential biomarkers that warrant investigation in OSA. Future studies should explore additional biomarkers to identify a comprehensive panel of biomarkers for OSA diagnosis and management.

Another potential limitation of our study is that we utilized polygraphy instead of polysomnography for sleep monitoring. This choice may have limited the number of parameters measured, such as EEG, EOG, and EMG, which are characteristic of polysomnography and could have provided more comprehensive sleep data.

Another key limitation of our study is the difference in BMI between the OSA group and the control group. While both groups were matched for age and sex, the OSA group had a higher average BMI compared with the control group. Given that BMI can influence systemic inflammation, which might affect the levels of the biomarkers we studied, this disparity could potentially introduce a confounding variable. Future studies should consider matching participants for BMI or statistically adjusting for BMI to better isolate the relationship between OSA and the studied biomarkers.

While there are noticeable trends in biomarker levels between the Moderate and Severe OSA groups, the lack of statistical significance suggests that these differences might not be substantial or might not hold clinical relevance. These observations might hint that the severity of OSA, as determined by AHI, may not significantly affect the levels of these biomarkers in plasma and serum. However, it is essential to acknowledge that the group sizes in this study might have been too small to detect a more subtle difference. Studies with larger sample sizes could provide a more nuanced understanding of the potential impact of OSA severity on these biomarker levels and their relevance in the pathophysiology and management of OSA.

Moreover, our study assessed biomarker concentrations at a single time point, which may not provide a complete understanding of the biomarkers’ roles in the pathophysiology of OSA.

One potential limitation of the study is that despite the efforts to control for confounding variables such as BMI, age, and gender in the multiple linear regression analysis, there were instances where significant coefficients were observed between biomarker levels and the OSA status, even though the study did not anticipate these relationships. This could be attributed to factors such as sample size, Type I error, unaccounted-for confounding variables, interaction effects, and statistical power. These unexpected significant coefficients highlight the complexity of interpreting regression results and underscore the need for cautious interpretation when examining associations between biomarkers and OSA status.

Lastly, prior to reaching any definite conclusions regarding the consideration of IL-6, IL-8, IL-10, TNF-α, CRP, and S100B as potential biomarkers for diagnosing moderate and severe OSA, our findings should be validated in a new validation cohort. Larger and more diverse cohorts, multi-center and longitudinal studies, and controlling for potential confounding factors are necessary to validate our findings and provide more robust estimates of the diagnostic performance of these biomarkers. Future research should also explore additional biomarkers, alternative diagnostic criteria, or a combination of multiple diagnostic methods, and standardize pre-analytical factors to ensure the reproducibility and comparability of results across studies.

## 4. Materials and Methods

### 4.1. Ethical Approval and Informed Consent

The study was approved by the Bioethics Committee of the Medical University of Bialystok (Poland; approval no. APK.002.207.2020) and conducted according to GCP/Guidelines for Good Clinical Practice. A signed written informed consent form and acceptance to participate in the study were received from all participants in the study.

### 4.2. Study Protocol and Subject Enrollment

The study was conducted within the Department of Otolaryngology from September 2020 to October 2022. Participants were either included or excluded based on specific criteria.

The OSA group participants were consecutive patients required from the Outpatient Clinic and the Department of Otolaryngology, were between the ages of 18 and 65, haD a documented moderate (15 ≤ AHI ˂ 30) or severe (AHI ≥ 30) OSA as per the home sleep study (PG), and provided signed written informed consent.

Patients were excluded if they had central sleep apnea syndrome; had extreme obesity (BMI ≥ 40); were on immune-suppressing therapy including inhaled, oral, or nasal steroids or other anti-inflammatory drugs within three months prior to the study; had undergone sleep apnea treatment within the three months before the study; had a history of respiratory infection within the previous four weeks; had a history of rheumatic diseases, coagulation disorders, acute or chronic kidney failure, recent injury, or surgery within the past three months; had severe cardiovascular disease, ischemic stroke, systemic inflammatory diseases, or any comorbidities that could potentially influence systemic inflammation such as: dermatitis, pulmonary disorders, especially asthma, or chronic otorhinolaryngological conditions that could result in elevation of inflammatory parameters, collagen vascular disease or cancer; or were taking hormones, immunosuppressants, or free radical scavengers.

The Control group consisted of non-OSA adults who did not exhibit OSA symptoms, did not snore, had no local or systemic inflammatory disease, and had AHI<5 as confirmed by a sleep study PG. Each patient was subjected to the same procedures as per the study protocol, which included taking a medical history, calculating BMI, performing an otorhinolaryngological examination, and conducting a PG.

### 4.3. Sleep Study

A portable sleep study device type III (SOMNOtouch, SOMNOmed) was used to conduct a polygraphy in each case. The following parameters were assessed during the PG for this study: Apnea-Hypopnea Index (AHI), mean oxygen saturation (MOS), lowest oxygen saturation (LSAT), and duration of sleep with blood oxygen saturation below 90% (SpO₂ < 90). The AHI represents the total number of apnea and hypopnea events per hour of sleep as recorded during an overnight sleep study. The severity of OSA can be evaluated through the AHI, which categorizes the condition into three levels: mild (5 ≤ AHI < 15), moderate (15 ≤ AHI ˂ 30), and severe (AHI ≥ 30) [35]. Apneas are characterized by a minimum of 90% reduction in airflow for at least 10 s, while hypopneas involve a decrease in respiratory signals for at least 10 s accompanied by a minimum of 3% oxygen desaturation [35]. An MOS typically ranging from 94% to 98% during sleep is considered normal [2].

### 4.4. Blood Sampling

Whole blood was collected from the subjects to obtain the serum and plasma for the evaluation of the concentrations of IL-6, IL-8, IL-10, TNF-alpha, CRP, and S100B. A fasting blood sample was gathered in the morning between 7–9 a.m. Blood for plasma samples was collected into two 2.7 mL tubes treated with the anticoagulant EDTA and was centrifuged immediately after collection. Blood for serum samples was collected into two 5.5 mL tubes with plastic granules with a coagulation activator and allowed to completely clot for 30 min at room temperature. A total of approximately 16.4 mL of venous blood was centrifuged at 2000 × *g* for 10 min at +4 °C, and the obtained serum and plasma were stored at −80 °C until the assays were performed.

### 4.5. Serum and Plasma Laboratory Testing

Serum levels of CRP were measured by the immunoturbidimetric method on an ALINITY CI analyzer (Abbott Laboratories, Abbott Park, IL, USA), while concentrations of S100B were examined using electrochemiluminescence immunoassay (ECLIA) on a cobas e411 analyzer. Luminex Discovery Assay multiplex kits designed for the Luminex 100/200™ were used to assess the levels of tested biomarkers. Assays were run in duplicate according to the manufacturer’s protocol in the Department of Neurodegeneration Diagnostics by the same researcher. Summarily, 50 µL of each sample was incubated with antibody-coated capture beads for 2 h at room temperature on a titer plate shaker. Washed beads were further incubated with biotin-labeled anti-human antibodies for 1 h at room temperature followed by incubation with streptavidin-phycoerythrin for 30 min and followed by an additional wash step. After the final wash, beads in the 96-well microtiter plate were resuspended in 100 μL wash buffer and loaded into the Luminex instrument. An acquisition gate was set between 8000 and 16,500 for the doublet discriminator, the sample volume was 50 μL, and 100 events/regions were acquired. Data analysis (mean fluorescence intensity) from all the bead combinations tested was performed using the xPONENT 4.3 quantification software (Luminex, Austin, TX, USA) due to calculating the sample concentrations. The sensitivity ranges for the biomarkers were as follows: CRP had a range of 0.10–48 mg/dL as measured using the Alinity apparatus, IL-6 had a range of 1.5–5000 pg/mL, and S100 had a range of 0.005–39 micrograms/L, both measured using the Cobas e411 apparatus. For the Luminex assay, the sensitivity ranges for specific biomarkers were as follows: IL-8/CXCL8 ranged from 1.23 to 900 pg/mL, IL-10 ranged from 1.43 to 1040 pg/mL, and TNF-alpha ranged from 2.09 to 1520 pg/mL. These sensitivity ranges were determined through rigorous calibration and validation procedures to ensure the accuracy and reliability of our measurements.

### 4.6. Statistical Analysis

Our statistical analysis was executed using GraphPad Prism 9. We initiated the Shapiro–Wilk test to assess the normality of data distribution. Given that the data were not normally distributed, we employed nonparametric tests for the analyses. The Mann–Whitney U test was utilized for comparisons across the groups.

In the case of biomarkers with concentrations falling below the detection limit in certain subjects, we employed a specific approach for handling such data. Values below the detection threshold were treated as equivalent to the detection threshold itself, as per the recommendation by Helsel [43]. The nonparametric rank–sum test appropriately treats all such values as tied at the lowest possible rank, accurately reflecting the uncertainty beneath the detection limit and avoiding the imposition of arbitrary values. In other statistical analyses conducted in this study, such as ROC curve analysis, Spearman correlation analysis, and regression models, this approach further reduced the probability of type 1 errors, thereby enhancing the robustness and reliability of our findings.

The chi-square test was utilized to calculate the difference between the study group and the control group regarding comorbidities and tobacco smoking. By employing this test, the proportions between the two groups could be compared, enabling the evaluation of significant differences.

We used Spearman correlation analysis to assess mutual dependencies between the concentrations of IL-6, IL-8, IL-10, TNF-α, CRP, and S100B in both serum and plasma and their correlations with OSA severity, sex, age, BMI, and AHI. Effect sizes for differences in the concentrations of these biomarkers in the serum and plasma across all subjects were evaluated using Hodges’ g statistic.

Receiver Operating Characteristic (ROC) curve analyses were computed to calculate the area under the curve (AUC) for sensitivity and specificity of IL-6, IL-8, TNF-α, CRP, and S100B in distinguishing between non-OSA controls and moderate-to-severe OSA subjects.

To further explore the associations, we incorporated multiple logistic regression analyses with OSA status as the dependent variable and the concentration of biomarkers as independent variables to assess their utility in classifying participants into OSA and non-OSA groups.

Multiple linear regression analyses were carried out to adjust for potential confounders, including BMI, age, and sex, thus providing a more robust estimation of the relationship between biomarker concentrations and OSA status. All statistical hypotheses were validated at a significance level of α = 0.05.

## 5. Conclusions

This research highlights the potential of CRP, S100B, IL-6, TNF-α, and IL-8 as serum and plasma biomarkers in diagnosing OSA, supplementing traditional methods such as overnight sleep studies. These biomarkers, found to be elevated in OSA patients, correlated positively with disease severity, age, and BMI. Future work should focus on the diagnostic and predictive power of these biomarkers in OSA management. Discerning whether diagnostic accuracy varies between serum and plasma concentrations of these biomarkers could lead to improved diagnostic procedures.

## Figures and Tables

**Table 1:** Basic demographic, anthropometric, sleep test parameters and comorbidities of the OSA and non-OSA participants of the study.

	OSA group (n = 52)	Control group (n = 28)	*p*	Moderate OSA group (n = 27)	*p* ^	Severe OSA group (n = 25)	*p* ”
**Sex male/female** **(% of male)**	44/8 (85%)	18/10 (64%)	0.1001	23/4 (85%)	0.2270	21/4 (84%)	0.1286
**Age [years]**	41; 21–65 (IQR 35–55)	37; 24–64 (IQR 29–52)	0.0628	37; 20–64 (IQR 34–53.50)	0.5006	37.5; 21–65 (IQR 33.5–57.8)	0.0761
**BMI [kg/m^2]^**	28.62; 21.02–39.31 (IQR 26.86–30.67)	26.53; 20.18–31.63 (IQR 25.00–28.93)	**0.0022**	28.52; 22.02–34.41 (IQR 27.66–30.92)	**0.0147**	30.56; 24.56–39.31 (IQR 28.05–34.58)	**0.0002**
**AHI [events/hour]**	28.30; 15.40–107.5 (IQR 19.90–43.90)	2.45; 0.1–4.9 (IQR 0.575–3.975)	**<0.0001**	19.45; 15.40–29.80 (IQR 15.90–24.33)	**<0.0001**	45.10; 31–107.5 (IQR 41.35–61.40)	**<0.0001**
**MOS [%]**	93; 70–96 (IQR 91.3–94)	96; 93–97 (IQR 95–97)	**<0.0001**	94; 88–96 (IQR 92.13–95)	**<0.0001**	93; 70–96 (IQR 90.5–93)	**<0.0001**
**LSAT [%]**	81; 35–92 (IQR 75–84)	92.5; 86–95 (IQR 91–93.25)	**<0.0001**	82; 71–92 (IQR 79.25–86)	**<0.0001**	75; 35–87 (IQR 65–82)	**<0.0001**
**SpO₂<90% [%]**	5.9; 0–84.5 (IQR 1.2–25.7)	0; 0–0.2 (IQR 0–0)	**<0.0001**	2.1; 0–75.9 (IQR 0.325–8.325)	**<0.0001**	24.4; 0.6–84.5 (IQR 9.8–41.5)	**<0.0001**
**Hypertension** **[n (%)]**	23 (44%)	6 (21%)	0.0531	6 (22%)	0.9681	17 (68%)	**0.0037**
**Comorbidities** **[n (%)]**	9 (17%)	2 (7%)	0.207	3 (11%)	0.971	6 (24%)	0.394
**Tobacco smokers** **[n (%)]**	15 (29%)	5(18%)	0.278	4 (15%)	0.760	11 (44%)	0.083

Values are presented as mean ± standard deviation—SD (in the case of normal distribution of the data), median and minimum-maximum (Interquartile range) (if there was no normal distribution of the data), or the number of subjects (percentage of the group). The Mann–Whitney U test was utilized for comparisons across the groups. Abbreviations: OSA, Obstructive Sleep Apnea; BMI, Body Mass Index; AHI, Apnea-Hypopnea Index; MOS, mean oxygen saturation; LSAT, lowest oxygen saturation; SpO2 < 90%, percentage of sleep spent with blood saturation <90%; *p*, *p*-value of the OSA group vs. the Control group; *p* ^, *p*-value of the Moderate OSA group vs. the Control Group; *p* ”, *p*-value of the Severe OSA group vs. the Control group IQR, Interquartile range; n, number of participants; bold emphasised values, statistically significant p-values; Comorbidities: diabetes mellitus, coronary artery disease, hyperuricemia.

**Table 2:** Correlations between the investigated parameters.

	IL-6	IL-8	IL-10	CRP	S100B	TNF-alpha	IL-6	IL-8	IL-10	CRP	S100B	TNF-alpha	OSA status	Sex	Age	BMI	AHI
	serum	serum	serum	serum	serum	serum	plasma	plasma	plasma	plasma	plasma	plasma					
IL-6 serum																	
IL-8 serum	NS																
IL-10 serum	0.279 *	NS															
CRP serum	0.496 ‡	NS	NS														
S100B serum	0.370 ‡	NS	0.262 *	NS													
TNF-alpha serum	0.231 *	NS	0.344 *	NS	NS												
IL-6 plasma	0.516 ‡	NS	NS	0.379 *	NS	NS											
IL-8 plasma	NS	0.306 *	NS	0.258 *	NS	0.287 *	0.315 *										
IL-10 plasma	NS	NS	0.740 ‡	NS	NS	NS	NS	NS									
CRP plasma	0.464 ‡	NS	NS	0.807 ‡	NS	0.256 *	0.344 *	NS	NS								
S100B plasma	NS	NS	NS	NS	−0222 *	NS	0.240 *	NS	NS	NS							
TNF-alpha plasma	0.325 *	NS	NS	0.273 *	NS	0.593 ‡	0.278 *	0.602 ‡	NS	0.305 *	NS						
OSA status	0.377 ‡	NS	NS	0.413 ‡	NS	0.305 *	NS	0.403 ‡	NS	0.387 ‡	−0.247 *	0.401 ‡					
Sex	NS	NS	NS	NS	NS	NS	NS	NS	NS	NS	NS	NS	0.001 ‡				
Age	0.262 *	NS	NS	NS	NS	NS	NS	0.313 *	NS	0.225 *	NS	NS	NS	NS			
BMI	0.291 *	0.300 *	NS	0.323 *	NS	0.254 *	NS	0.261 *	NS	0.300 *	NS	0.322 *	0.231 *	NS	0.258 *		
AHI	0.310 *	NS	NS	0.481 ‡	NS	0.228 *	NS	0.418 ‡	NS	0.485 ‡	−0.286	0.341 *	0.814 ‡	0.375 ‡	0.287 *	0.359 ‡	

The results of Spearman correlation analysis are expressed as r values and the level of statistical significance (*p*). Numbers presented in the table show the correlation coefficients, and the signs indicate the statistical differences at the two significance levels. The values of r with *p* < 0.05 were considered statistically significant (* *p* < 0.05, ‡ *p* < 0.001). Abbreviations: NS, not statistically significant (*p* > 0.05); IL-6, interleukin-6; IL-8, interleukin-8; IL-10, interleukin-10; CRP, C-reactive protein; S100B, S100 calcium-binding protein B; TNF-alpha, Tumor Necrosis Factor alpha; OSA, Obstructive Sleep Apnea; BMI, Body Mass Index; AHI, Apnea-Hypopnea Index.

**Table 3:** Calculated medians, minimum-maximum values, and interquartile ranges, mean and standard deviations of the concentrations of IL-6, IL-8, IL-10, TNF-α, CRP, and S100B in serum and plasma in Moderate and Severe OSA groups.

		Moderate OSA	Severe OSA	*p*
Serum	CRP [pg/mL]	1.2; 0.9–38.9 (0.975–3.125)	1.9; 0.9–117.6 (1.3–4.7)	0.0922
	S100B [pg/mL]	0.0415; 0.023–0.203 (0.0355–0.05225)	0.046; 0.02–0.094 (0.034–0.059)	0.5162
	IL-6 [pg/mL]	3.065; 1.4–58.57 (1.5–9.193)	2.23; 1.4–20 (1.5–4.05)	0.2733
	TNF-alpha [pg/mL]	3.86; 2.04–5.59 (2.543–4.678)	3.72; 1.48–8.77 (2.82–4.85)	0.7340
	IL-8 [pg/mL]	8.165; 1.87–15.17 (6.613–9.635)	9.93; 3.63–38.86 (7.49–12.44)	0.0619
	IL-10 [pg/mL]	2.04; 0.71–103.5 (1.4–33.9)	1.43; 1.399–92.14 (1.4–9.62)	0.6187
Plasma	CRP [pg/mL]	1.6; <1–114 (<1–3.3)	1.8; <1–114 (<1–113)	0.0559
	S100B [pg/mL]	<0.005; <0.004–0.049 (<0.004–0.045)	<0.005; 0.004–0.009 (0.004–0.005);	0.0736
	IL-6 [pg/mL]	<1.5; <1.5–24.5 (1.4–23.1)	<1.5; <1.5–16.9 (<1.5–15.5)	0.6204
	TNF-alpha [pg/mL]	3.09; 1.69–8.9 (1.69–7.21)	3.73; 1.25–8.07 (1.25–6.82)	0.5348
	IL-8 [pg/mL]	4.2; 1.23–9.41 (1.23–8.18)	4.98; 1.35–14 (1.35–12.7)	0.1423
	IL-10 [pg/mL]	<1.43; <1.43–6.49 (<1.43–5.09)	<1.4; <1.4–7.38 (<1.4–5.98)	0.4525

Values are presented as median; minimum–maximum (Interquartile range). Values represented with the “<“ sign indicate the detection threshold of studied biomarkers. The Mann–Whitney U test was utilized for comparisons across the groups, with *p* < 0.05 considered statistically significant. Abbreviations: IL-6, Interleukin-6; IL-8, Interleukin-8; IL-10, Interleukin-10; CRP, C-reactive protein; TNF, Tumor Necrosis Factor; S100B, S100 calcium-binding protein B; OSA, Obstructive Sleep Apnea.

**Table 4:** Multiple linear regression analyses coefficients IL-6, IL-8, TNF-α, CRP, and S100B in the serum and plasma.

		OSA Status	*p*	Sex	*p*	Age	*p*	BMI	*p*
Serum	CRP	3.489	0.377	2.911	0.5452	0.2447	0.0693	−0.1389	0.7726
	IL-6	4.314	0.056	−1.682	0.5369	0.1184	0.1193	0.2644	0.3327
	TNF-α	0.6883	0.1478	−0.2836	0.6229	0.001582	0.9211	0.05434	0.3472
Plasma	CRP	3.327	0.3768	2.731	0.5573	0.2361	0.0698	−0.118	0.7979
	TNF-α	1.912	**0.0016**	−0.7436	0.2021	0.01265	0.4345	0.1251	0.0543
	IL-6	1.369	0.2854	−0.9693	0.5404	0.1595	**0.0005**	−0.02339	0.8813
	IL-8	1.46	**0.0273**	0.1184	0.8832	0.0381	0.0907	0.1241	0.1231
	S100B	0.0005	0.7376	0.0014	0.4741	0.0001	0.1009	−0.0002	0.4474

Values are presented as P value of multiple linear regression coefficients, *p* < 0.05 considered statistically significant. Abbreviations: IL-6, Interleukin-6; IL-8, Interleukin-8; IL-10, Interleukin-10; CRP, C-reactive protein; TNF, Tumor Necrosis Factor; S100B, S100 calcium-binding protein B; BMI, Body Mass Index.

## Data Availability

The data supporting this study’s findings are available on request from the corresponding author.

## Supplementary Materials

The supporting information can be downloaded at: https://www.mdpi.com/article/10.3390/ijms241813875/s1.
