# Supplementary material for: Investigating the Role of Serum and Plasma IL-6, IL-8, IL-10, TNF-alpha, CRP, and S100B Concentrations in Obstructive Sleep Apnea Diagnosis
Source: Int J Mol Sci. 2023 Sep 9;24(18):13875. doi: 10.3390/ijms241813875 (PMC10530258; doi:10.3390/ijms241813875)
Supplement: Supplementary file 1 [file ijms-24-13875-s001.zip › ijms-2554870-supplementary.pdf]

Supplementary Material - Table S1.– The concentrations of IL-6, IL-8, IL-10, TNF-α, CRP, and S100B in the serum and plasma

		OSA group (n=52)	Control group (n=28)	Effect size *Hedges' g*
**Serum**	**IL-6 [pg/ml]**	2.29; <1.5-58.6 (1.5-6.36)	<1.5;<1.5-10.2 (<1.5-<1.5)	0.52
	**IL-8 [pg/ml]**	8.83; 1.87-38.9 (6.81-11.2)	6.72; 2.07-20.6 (4.6-11.3)	0.61
	**IL-10 [pg/ml]**	<1.43; 0.71-104 (<1.43-10.5)	<1.43; <1.43-12.3 (<1.43-2.13)	0.57
	**CRP [pg/ml]**	1.7; <1-118 (<1-3.9)	<1; <1-5 (<1-<1)	0.44
	**TNF-α [pg/ml]**	3.72; 1.48-8.77 (2.82-4.74)	2.82; 0.62-12.3 (2.04-4.16)	0.36
	**S100B [pg/ml]**	0.045; 0.02-0.203 (0.035-0.058)	0.038; 0.017-0.081 (0.03-0.049)	0.36
**Plasma**	**IL-6 [pg/ml]**	<1.5; <1.5-24.5 (<1.5-4.17)	<1.5; <1.5-20.9 (<1.5-1.54)	0.16
	**IL-8 [pg/ml]**	4.8; <1.23-14 (3.51-6.63)	2.84; <1.23-9.65 (1.71-4.8)	0.82
	**IL-10 [pg/ml]**	<1.43; <1.43-7.38 (<1.43-1.44)	<1.43; <1.43-1.63 (<1.43-1.43)	0.54
	**CRP [pg/ml]**	1.6; <1-114 (<1-3.3)	<1; <1-4.9 (<1-<1)	0.37
	**TNF-α [pg/ml]**	3.21; <2.09-8.9 (2.66-5.16)	<2.09; <2.09-5.51 (<2.09-3.68)	0.46
	**S100B [pg/ml]**	<0.005; <0.005-0.049 (<0.005 <0.005)	<0.005;< 0.005-0.006 (<0.005-<0.005)	0.20

Values are presented as median; minimum-maximum (Interquartile range). Values represented with the "<" sign indicate the detection threshold of studied biomarkers. Abbreviations: IL-6, Interleukin-6; IL-8, Interleukin-8; IL-10, Interleukin-10; CRP, C-reactive protein; TNF, Tumor Necrosis Factor; S100B, S100 calcium-binding protein B; OSA, Obstructive Sleep Apnea.
